# Exosome-based strategy for degenerative disease in orthopedics: Recent progress and perspectives

**DOI:** 10.1016/j.jot.2022.05.009

**Published:** 2022-07-11

**Authors:** Rongjie Wu, Haotao Li, Chuanwei Sun, Jialin Liu, Duanyong Chen, Haiyang Yu, Zena Huang, Sien Lin, Yuanfeng Chen, Qiujian Zheng

**Affiliations:** a Department of Orthopedics, Guangdong Provincial People’s Hospital, Guangdong Academy of Medical Sciences, Guangzhou, PR China; b Shantou University Medical College, Shantou, China; c Research Department of Medical Science, Guangdong Provincial People’s Hospital, Guangdong Academy of Medical Sciences, Guangzhou, PR China; d Department of Burn and Wound Repair Surgery and Research Department of Medical Science, Guangdong Provincial People’s Hospital, Guangdong Academy of Medical Sciences, Guangzhou, PR China; e Rehabilitation Center, Shengjing Hospital Affiliated to China Medical University, Shenyang, Liaoning, PR China; f Department of General Medicine, Guangdong Provincial People’s Hospital, Guangdong Academy of Medical Sciences, Guangzhou, China; g Department of Orthopaedics & Traumatology, Stem Cells and Regenerative Medicine Laboratory, Li Ka Shing Institute of Health Sciences, The Chinese University of Hong Kong, Prince of Wales Hospital, Shatin, Hong Kong Special Administrative Region of China; h Southern Medical University, Guangzhou, PR China

**Keywords:** Degenerative disease, Orthopaedics, Exosome

## Abstract

**Background:**

Degenerative diseases in orthopaedics have become a significant global public health issue with the aging of the population worldwide. The traditional medical interventions, including physical therapy, pharmacological therapy and even surgery, hardly work to modify degenerative progression. Stem cell–based therapy is widely accepted to treat degenerative orthopaedic disease effectively but possesses several limitations, such as the need for strict monitoring of production and storage and the potential risks of tumorigenicity and immune rejection in clinical translation. Furthermore, the ethical issues surrounding the acquisition of embryonic stem cells are also broadly concerned. Exosome-based therapy has rapidly grown in popularity in recent years and is regarded as an ideal alternative to stem cell–based therapy, offering a promise to achieve ‘cell-free’ tissue regeneration.

**Methods:**

Traditionally, the native exosomes extracted from stem cells are directly injected into the injured site to promote tissue regeneration. Recently, several modified exosome–based strategies were developed to overcome the limitations of native exosomes, which include mainly exogenous molecule loading and exosome delivery through scaffolds. In this paper, a systematic review of the exosome-based strategy for degenerative disease in orthopaedics is presented.

**Results:**

Treatment strategies based on the native exosomes are effective but with several disadvantages such as rapid diffusion and insufficient and fluctuating functional contents. The modified exosome–based strategies can better match the requirements of the regeneration in some complex healing processes.

**Conclusion:**

Exosome-based strategies hold promise to manage degenerative disease in orthopaedics prior to patients reaching the advanced stage of disease in the future. The timely summary and highlights offered herein could provide a research perspective to promote the development of exosome-based therapy, facilitating the clinical translation of exosomes in orthopaedics.

**Translational potential of this article:**

Exosome-based therapy is superior in anti-senescence and anti-inflammatory effects and possesses lower risks of tumorigenicity and immune rejection relative to stem cell–based therapy. Exosome-based therapy is regarded as an ideal alternative to stem cell–based therapy, offering a promise to achieve ‘cell-free’ tissue regeneration.

## 1 Introduction

With the aging of the population worldwide, degenerative diseases in orthopaedics have become a major global public health issue that urgently require a solution. Osteoarthritis (OA) and intervertebral disc degeneration (IVDD) are the most common degenerative orthopaedic disorders, which cause pain and disability, leading to increased social burden among the expanding and aging population [[1], [2], [3]]. A substantial number of medical interventions, including physical therapy, pharmacological therapy and even surgery, have been investigated to slow or halt the degenerative process; however, these strategies provide limited benefits for modifying degenerative progression. In recent years, rapidly developed stem cell therapies have emerged as treatment candidates with the most potential to reverse the degenerative process owing to their capacity for restoring tissue and modulating inflammation [[4], [5], [6]]. However, some limitations, such as the need to strictly monitor production and storage and the potential risks of tumorigenicity and possible immune rejection, have greatly limited the clinical translation of stem cell–based tissue engineering therapy [[7], [8], [9], [10], [11]]. In addition, the ethical controversies surrounding the acquisition of embryonic stem cells have also restricted their use [12,13].

With the progression of research, extracellular vesicles (EVs) were recently demonstrated to be the primary functional molecules involved in tissue regeneration mediated by stem cells [14,15]. It was reported that EVs alone could duplicate the therapeutic power of stem cells in a number of animal disease models, providing the possibility of developing a novel therapy for degenerative orthopaedic diseases [16,17]. EVs are composed of two subtypes: endosome-origin ‘exosomes’ and plasma membrane–derived ‘ectosomes’ [18]. Broadly, EVs are particles released from cells to deliver signals to specific cells and can be separated by various techniques, including differential ultracentrifugation, density gradients, precipitation, filtration, size-exclusion chromatography and immuno-isolation [19]. However, EVs including exosomes prepared by current protocols are heterogeneous, with an absence of demonstrated purity and origin due to their overlapping sizes and lack of specific markers [20]. Thus, the term ‘exosome’ is often used in the literature to refer to small EVs able to pass through 220-nm pore filters or to be recovered by high-speed ultracentrifugation [21]. The native exosomes secreted by stem cells are proven to have excellent anti-senescence and anti-inflammatory effects. Additionally, the lower risks of tumorigenicity and immune rejection allow exosome-based therapy to be an alternative to stem cell transplantation in degenerative diseases. The lower requirement of exosome storage compared to stem cell storage could also facilitate their transportation and further application.

In addition to the intrinsic effect exerted by endogenous substances, exosomes are a promising vehicle with diameters around 30–150 ​nm, enabling the delivery of exogenous bioactive molecules like microRNAs (miRNAs), proteins and small molecules to specific cells [22,23]. With their bilayer phospholipid and nano-sized structures, exosomes can pass through various biological barriers without cargo wastage [24]. Given the endogenous sources of exosomes, it is also believed that the minimal tumorigenic risk will increase while using exosomes as the vehicle for delivering

Small molecule drugs [25,26]. Currently, exosomes are mostly used in the orthopaedic field for the management of OA and IVDD, the leading degenerative diseases contributing to disability in older individuals. In this review, we mainly focused on the application of exosomes in their native status or as a vehicle for delivering functional substances to treat OA and IVDD, providing a comprehensive view of the recent developments in exosome-based strategies for degenerative disease in orthopaedics.

## 2 Exosome-based strategies for OA

### 2.1 Native exosome applications in OA

#### 2.1.1 Promotion of cell proliferation and migration

**Fig. 1:**
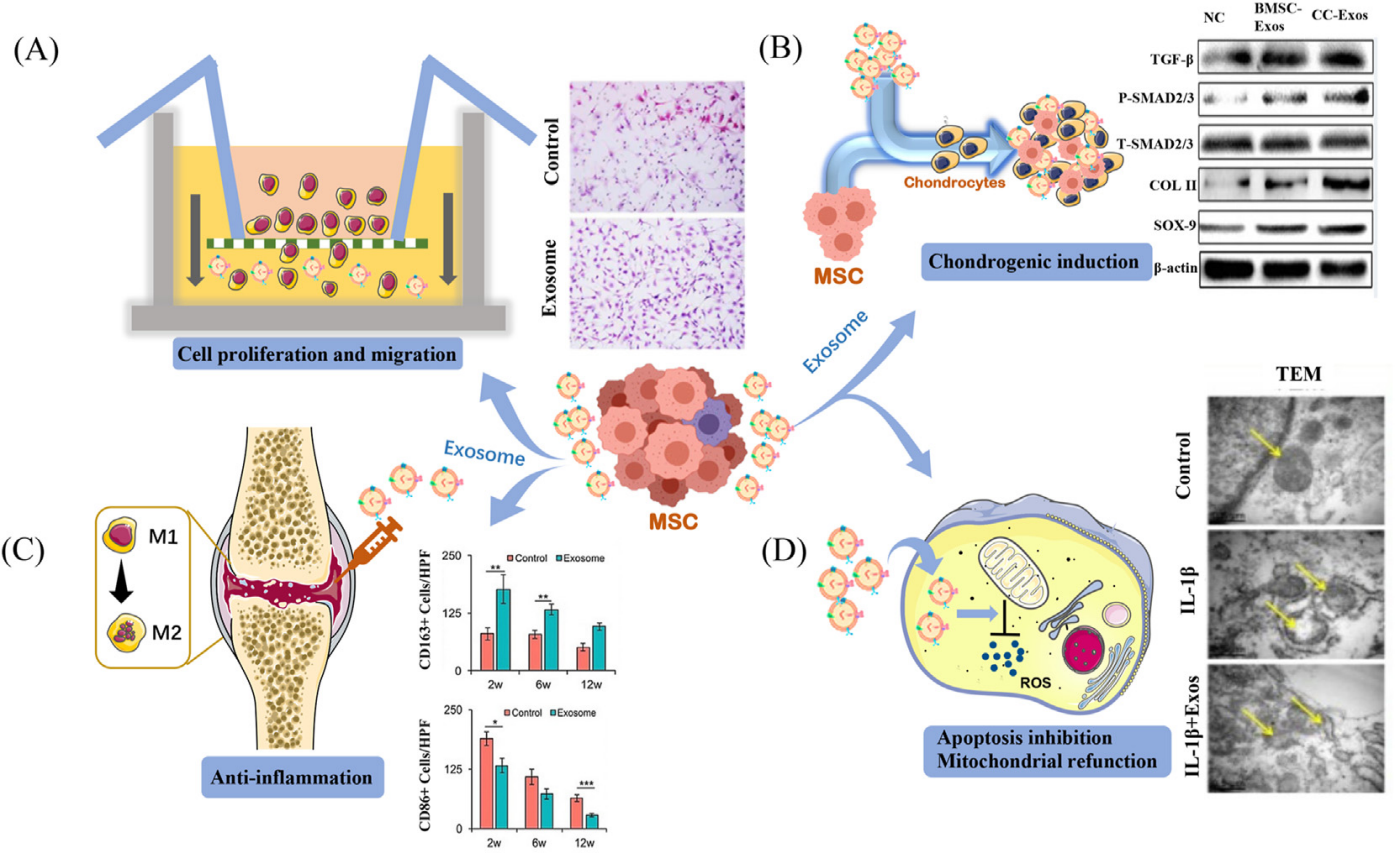
Illustration of the functions that native exosomes exert in osteoarthritis. (A) Cell proliferation and migration. (B) Chondrogenic induction. (C) Anti-inflammation. (D) Apoptosis inhibition and mitochondrial refunction; the yellow arrows indicate mitochondria. [Reproduced with permission from ref 17. Copyright 2018 Elsevier; ref 23. Copyright 2018 TAYLOR & FRANCIS; ref 31. Copyright 2019 Ivyspring International Publisher.].

Previous studies have revealed that co-culturing chondrocytes with stem cells successfully prompted cartilage matrix production and repair, which was explained by the promotional effect of mesenchymal stem cell (MSC) secretion on aged chondrocyte proliferation [27,28]. Currently, exosomes have been marked as one of the most important components in paracrine secretion, providing regenerative promotion to endogenous cells and reactivating matrix restoration in aging tissue. The cartilage regeneration capacity of MSC-derived exosomes (MSC-Exos) was partially attributed to the facilitation of chondrocyte migration (Fig. 1A), as Zhang et al. indicated that MSC-Exos are available to directly and rapidly communicate with chondrocytes through endocytosis, facilitating endogenous chondrocyte migration towards defects both *in vitro and in vivo* [11,29]. In addition to enhanced migration, the chondrocytes treated by MSC-Exos also displayed improved cell proliferation as evidence of increased metabolic activity and DNA contents compared to those that remained untreated [11,29]. Although MSC-Exos are widely accepted to support the proliferation and migration of endogenous chondrocytes, the specific molecular mechanism has remained under investigation. Some researchers suggested that miRNAs such as miR-136-5p play an essential role in accelerating chondrocyte proliferation and migration [30]. Nevertheless, other studies also indicate that long non-coding RNAs and proteins within exosomes are effective in eliciting rapid cellular proliferation and migration. For instance, exosomal CD73 or the long non-coding RNA H19 was reported to make a contribution to rapid chondrocyte proliferation and infiltration in exosome-mediated cartilage repair [11,31]. In addition to recruiting chondrocytes, it was also reported that MSC-Exos can promote the migration and proliferation of MSCs to replenish the active stem cells available for cartilage regeneration [32,33]. To clarify the functional components mediating the therapeutic effect of native exosomes in OA and their relevant molecular pathways, we have summarised them in Table 1.

**Table 1:** Summary of the functional components mediating therapeutic effect of native exosomes in OA and IVDD and the pathway regulated by each functional component.

Function of native exosomes	Cell sources	Function components	Regulatory pathways	Rf
**Exosomes for OA**				
Promotion of cells proliferation and migration	hESCs-MSCs	CD73	AKT and ERK signalling activation	[28]
	BMSCs	miR-136-5p	E74-like factor 3	[30]
	hUC-MSCs	LncRNA H19	unmentioned	[31]
	hMSCs	LncRNA-KLF3-AS1	miR-206/GIT1 axis	[34]
Chondrogenic induction	Chondrocytes	miR-8485	Wnt/beta-catenin pathways	[36]
Anti-inflammation	hESCs-MSCs, BMSCs	unmentioned	Increased M2 macrophages, decreased M1 macrophages	[28,39]
	BMSCs	LncRNA LYRM4-AS1	GRPR-miR-6515-5p	[42]
Apoptosis inhibition	IPFP-MSCs	miR-100-5p	mTOR-autophagy pathway inhibition	[43]
	hMSCs	LncRNA-KLF3-AS1	miR-206/GIT1 axis	[34]
Mitochondrial refunction	BMSCs	unmentioned	p38, ERK, and Akt pathways	[46]
**Exosomes for IVDD**				
NPCs proliferation and antisenescence	USCs	matrilin-3	TGF-β activation	[69]
Stem cells migration and differentiation	CESCs	unmentioned	HIF-1α/Wnt signaling activation	[70]
Refunction of cartilage endplate	CESCs	unmentioned	PI3K/AKT/autophagy pathway activation	[72]
	MSCs	miR-31-5p	ATF6-related endoplasmic reticulum stress regulation	[73]
Anti-inflammation	MSCs	unmentioned	NLRP3 inflammasome reduction	[78]
Anti-pyroptosis	MSCs	miR-410	NLRP3 pathway suppression	[79]
	hucMSC	miR-26a-5p	METTL14/NLRP3 pathway	[81]
Anti-apoptosis	MSCs	miR-21	PTEN-PI3K-Akt pathway silencing	[82]
	MSCs	miR-142-3p	MAPK signaling pathway suppression	[83]
	BMSCs, USCs		AKT and ERK signaling activation	[84,85]
Anti-angiogenesis	NCs	miR-140-5p	Wnt/β-catenin pathway	[87]

Abbreviation: OA, osteoarthritis; MSCs, mesenchymal stem cells, hESCs-MSCs, human embryonic stem cell-derived MSCs, hUC-MSCs, human umbilical cord mesenchymal stem cells; BMSCs, bone marrow mesenchymal stem cells; IPFP-MSCs, infrapatellar fat pad MSCs; IVDD, intervertebral disc degeneration; USCs, human urine-derived stem cells; CESCs, cartilage endplate stem cells; hucMSCs, human umbilical cord mesenchymal stem cells; NCs, notochordal cells.

#### 2.1.2 Chondrogenic induction

Increased proliferation of chondrocytes is usually accompanied by cell de-differentiation, leading to a disorientation of phenotypes and reduced cartilage production. However, chondrocytes treated by MSC-Exos remain available to maintain their phenotypes while presenting superior matrix production and chondrogenic gene expression (Fig. 1B) [34]. This excellent phenotype preservation can be attributed to the superior capacity of exosomes in guiding cells towards a chondrogenic linage. Liu et al. demonstrated that MSC-Exos could up-regulate chondrogenic genes such as *Col2a1* and aggrecan while down-regulating chondrocyte hypertrophy markers, including matrix metalloproteinase 13 and runt-related transcription factor 2, in chondrocytes from an OA model [34]. In addition to MSC-Exos, exosomes derived from mature chondrocytes are also available to promote cartilage progenitor cell proliferation and chondrogenic differentiation as evidenced by increased levels of messenger RNA and proteins like SOX-9 and COL II in cartilage progenitor cells *in vitro* and *in vivo* [35]. Remarkably, chondrocyte-derived exosomes are more effective in inhibiting chondrogenic hypertrophy—and consequently, calcification—compared to MSC-Exos and therefore better at regenerating high-quality cartilage with minimal hypertrophy and vessel ingrowth [35]. The exosomes from chondrocytes also held promise to promote chondrogenic differentiation of bone marrow mesenchymal stem cells (BMSCs), which was attributed to Wnt/β-catenin pathway activation [36] (Table 1).

#### 2.1.3 Anti-inflammation

It has been reported that aging-related inflammation is an important contributing factor to the development of OA [37]. Inflammatory mediators increase with age, destroying joint tissue and eventually leading to OA. Thus, anti-inflammation constitutes an essential component of tissue-regenerative strategies in OA, which could not only protect the spare cartilage but also provide a suitable environment for tissue regeneration. MSC-Exos were demonstrated to attenuate the inflammatory process and protect chondrocytes from degeneration (Fig. 1C). Cosenza et al. reported that MSC-Exos could reduce the local inflammation by increasing M2 macrophage infiltration with a concomitant reduction in M1 macrophages [38,39]. In addition, MSC-Exos were available to up-regulate the expression of anti-inflammatory interleukin (IL)-10 and transforming growth factor β1 while reducing the expression of pro-inflammatory IL-1β, IL-6, tumour necrosis factor (TNF)-α and IL12P40 [40]. Exosomal miRNAs are also considered to play the most important role in the anti-inflammatory effects on OA. For instance, miR-361-5p delivered by MSC-Exos has been demonstrated to alleviate IL-1β chondrocyte damage by inhibiting the nuclear factor κB signalling pathway [41]. A recent study also illustrated that the anti-inflammatory effect could be attributed to the competing endogenous RNA network as LYRM4-AS1-GRPR-miR-6515-5p [42]. Collectively, MSC-Exos could attenuate the inflammation closely related to OA, promising to protect aging joint tissue and creating a favourable microenvironment for chondrogenesis.

#### 2.1.4 Apoptosis inhibition and mitochondrial refunction

In addition to their anti-inflammatory effect, the inhibition of preserved cell apoptosis by exosomes is also indispensable to restoring tissue in the circumstances of OA (Fig. 1D). Zhang et al. found that cleaved caspase-3–positive apoptotic cells significantly decreased in number and proliferative cell nuclear antigen–positive cells increased in MSC-Exo–treated cartilage lesions compared to those not treated after 6 weeks of incubation [11]. Infrapatellar fat pad MSC–derived exosomes could also reduce chondrocyte apoptosis and protect cartilage from damage in OA, a phenomenon which may be attributed to an abundancy of miR-100-5p [43]. Moreover, cell apoptosis in degenerative diseases may be associated with mitochondrial dysfunction. Mitochondria are the energy-producing organelles involved in many critical biological processes, such as cell proliferation. The degenerative status of patients with OA can disturb mitochondrial respiration and overproduce reactive oxygen species, leading to mitochondrial dysfunction and oxidative stress damage in tissue-specific cells like chondrocytes [44]. Recently, Chen et al. found that MSC-Exos could repair mitochondrial dysfunction in OA by providing mitochondrial-related proteins to the degenerative cartilage and overcoming the insufficient energy supplementation [45]. Another study revealed that MSC-Exos could counter the mitochondrial membrane potential change of chondrocytes and consequently inhibit the mitochondrial dysfunction–induced apoptosis in OA [46].

### 2.2 Modified exosome–based therapy for OA

#### 2.2.1 Exosomes as a vehicle of exogenous miRNA

**Fig. 2:**
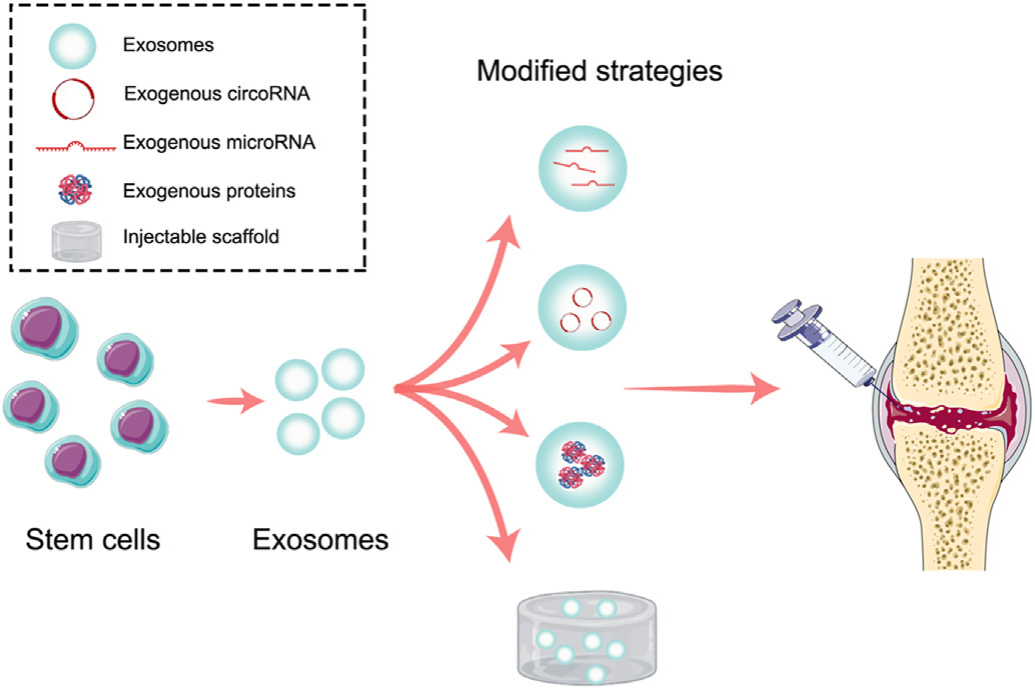
Schematic diagram of modified exosome–based therapy for degenerative osteoarthritis.

It has been widely accepted that the regenerative and anti-inflammatory effects exerted by exosomes are mainly attributable to small molecules, such as miRNAs and proteins, within exosomes. However, the contents of essential miRNAs within native exosomes derived from stem cells cannot fully match the requirements in some complex healing processes; the fluctuated contents of exosomes also limit their extensive application [47]. To overcome shortages of native exosomes, loading native exosomes with exogenous miRNA to benefit patients with OA may be a promising strategy (Fig. 2). Recently, engineered stem cells with specific miRNA-overexpression profiles have become attractive and commonly used to imbue exosomes with specific and stable functional miRNAs. For instance, Mao et al. prepared exosomes derived from miR-92a-3p–overexpressing MSCs, proposing to target WNT5A, the critical protein involved in OA development [48]. Their results illustrated MSC-Exos with abundant miR-92a-3p are able to promote chondrogenic differentiation and cartilage matrix synthesis by inhibiting WNT5A expression, showing promise to delay cartilage degradation in an OA mouse model [48].

Although native exosomes from synovial MSCs (SMSC-Exos) could also attenuate inflammation and promote the proliferation of chondrocytes, their inhibition of chondrocyte maturation restricted the capacity for cartilage restoration in OA. To overcome the shortage of native SMSC-Exos, Tao et al. overexpressed miR-140-5p in SMSCs and developed modified exosomes with an abundancy of miR-140-5p, an miRNA able to effectively promote chondrogenic differentiation of MSCs [49]. Compared to native SMSC-Exos, SMSC-Exos with exogenous miR-140-5p were able to maintain the function of chondrocytes while preserving the stimulation of cell proliferation and migration *in vitro*. In an OA mouse model induced by knee disability, the cartilage matrix composed of type II collagen was significantly greater in SMSC-140-Exos, demonstrating that modified miR-140-5p–enriched exosomes could be outstanding candidates for preventing OA progression. Apart from producing exosomes with an enhanced capacity to promote cell proliferation, engineered cells also hold promise to provide exosomes that effectively improve the microenvironment and protect the spare chondrocytes. Zhou et al. found that miR-126-3p, which plays a role in cell aging and senescence, was reduced in exosomes from OA patients [50]. In their study, synovial fibroblasts were transfected with miRNA-126-3p mimic to provide exosomes with miRNA-126-3p to degenerative chondrocytes and cartilage tissue in OA mice. The exosome-delivered miRNA-126-3p effectively suppressed cartilage degeneration and prevented OA progression *in vitro* and *in vivo*, a result which was attributed to the anti-apoptotic and anti-inflammatory effects [50].

In addition to their extraction from engineered MSCs, miRNA-enrich exosomes could also be produced by directly loading the native exosomes with functional exogenous miRNAs. For example, Tao et al. incorporated miR-361-5p into MSC-Exos through electroporation. In an *in vitro* study, the exosomal miR-361-5p targeting Asp-Glu-Ala-Asp-box polypeptide 20, the critical factor up-regulated in OA, showed superior performance in alleviating chondrocyte damage and inflammation induced by IL-1β. Furthermore, in an OA mouse model, reduced inflammation and synovial tissue hyperplasia were observed with the administration of exosomes with exogenous miR-361-5p [41]. In addition to electroporation, the freeze and thaw method was also used to incorporate miRNA (miR-140) into purified exosomes from plasma to construct an RNA delivery system (EX-miRNA-140), aiming to improve the chondrogenic regenerative effect by inducing membrane fusion followed by miRNA release into the cytoplasm. In an *in vitro* study employing MSCs, significantly higher cell-adhesion and -proliferation rates were revealed in the EX-miRNA-140 group relative to using miR-140 or exosomes individually. Enhanced cartilage matrix production and expression of chondrogenic genes like *SOX9* and aggrecan were also observed, indicating that EX-miRNA-140 effectively guided stem cells towards a chondrogenic lineage [51].

#### 2.2.2 Exosomes as a vehicle of exogenous circRNA

Although several miRNAs carried by exosomes have been shown to prevent or slow the progression of OA, their shortages of insufficient stability and their rapid clearance have reduced the enthusiasm for their further application [52]. Circular RNAs (circRNAs), as a class of covalently closed single-stranded circularised RNA molecules, possess the advantages of excellent environmental resistance and stability, rendering them promising alternatives to miRNAs to be the cargo of exosomes [53] (Fig. 2). Recent studies performing exosomal circRNA deep sequencing found that exosomal circRNA_0001236 plays an important role in the chondrogenic induction of MSCs [54]. Subsequently, researchers constructed MSC-Exos with abundant circRNA_0001236 by overexpressing circRNA_0001236 in MSCs and successfully attenuated structure damage and restored cartilage in OA [54]. Further, sleep-related circRNAs (e.g., circRNA3503) were identified to inhibit chondrocyte apoptosis in OA. Compared to applying SMSC-Exos or circRNA3503 individually, delivering circRNA3503 through SMSC-Exo application achieved better cartilage protection in OA through the synergistic effect of the extracellular matrix (ECM) synthesis promotion contributed by circRNA3503 and the ECM degradation inhibition contributed by SMSC-Exos [55]. Collectively, circRNAs may be an optimal cargo for native exosomes to resolve the problem of an insufficient regenerative ability. Furthermore, the exosome is a favourable vehicle that enables circRNA transportation to cartilage defects with maximal efficacy.

#### 2.2.3 Exosomes as the vehicle of protein

Exosomes are also able to provide an ideal platform for small molecular protein delivery to promote cartilage regeneration in OA (Fig. 2). The poor structural, chemical, and functional characteristics of regenerative cartilage remain tricky problems in exosome therapy due to insufficient chondrogenic differentiation. Kartogenin (KGN) is a recently discovered cytokine with a favourable chondrogenic effect but a shortage of poor water solubility, restricting its use in cartilage repair. Given that exosomes are an excellent vehicle for hydrophobic proteins, Xu et al. attempted to load KGN into E7-Exos targeting MSCs and developed a novel delivery vehicle with the advantage of reduced KGN aggregation due to hydrophobicity [24]. As expected, directly adding KGN into MSC culture medium led to aggregation inside cells, while KGN delivered by E7-Exos showed good distribution in the cytoplasm and effectively promoted cartilage matrix formation *in vitro*. The superior KNG delivery to MSCs could be attributed to the lipid membrane of exosomes, which contributed to the preservation and release of KGN. In an established rat OA model, KGN-E7-Exos showed a superior cartilage-regeneration effect with a possible anti-inflammatory ability, promising a potential new approach to cartilage reversion in OA [24].

WNT3a, a strong activator of the WNT/β-catenin pathway essential in cartilage repair, has been incorporated into exosomes to penetrate dense cartilage and deliver molecules deep into chondrocytes. However, WNT3a is an insoluble molecule and easily degraded at the injury site. As WNT3a has a high affinity with exosomes, exosomes from L-cells (ATCC CRL2647/CRL2648; American Type Culture Collection, Manassas, VA, USA) were employed to deliver WNT3a to prolong the effect and were injected into mouse joints to repair large osteochondral defects. After 8 weeks, WNT3a-loaded MSC-Exos had successfully improved osteochondral defect repair via WNT signalling activation, while WNT3a without vehicle failed to activate WNT signalling to promote cartilage regeneration [56]. Overall, exosomes are potential vehicles with good cartilage penetration and cargo preservation, promising the delivery of proteins and small molecules, especially for hydrophobic substances. In the future, proteins for stem cell recruitment, such as stromal cell-derived factor-1, could also be an alternative cargo of exosomes, further improving cell migration, which is critical for regenerative processes *in vivo* [57,58]. Furthermore, loading anti-inflammatory and chondrogenic proteins into exosomes simultaneously also holds promise to enhance cartilage regeneration synergistically [59].

#### 2.3 Cell-specific exosomes as a vehicle

Although exosomes provide an ideal platform for cargo delivery, the non-selectivity of native exosomes decreases the efficacy of cargo delivery to the target cells *in vivo*. Additionally, reticuloendothelial organs or macrophages might metabolise the exosomes before they are taken by chondrocytes or MSCs [60,61]. To promote the uptake of exosomes by synovial membrane–derived MSCs (SM-MSCs), Xu et al. installed an MSC-targeting sequence on exosomes (E7-Exo) to improve the efficiency of KGN delivery [24]. In an *in vitro* study employing SM-MSCs and chondrocytes, more fluorescent-labelled KGN was found in SM-MSCs, indicating that E7-Exos could recognise SM-MSCs and increase the efficiency of KGN delivery. Compared to KGN-loaded native exosomes, the delivery of KGN via E7-Exos also provoked better cartilage regeneration *in vivo.* Exosomes targeting chondrocytes are also critical to delivering cargos to chondrocytes precisely and effectively as the avascularity and high density of cartilage hinder transport efficiency. In order to increase the uptake of exosomes with functional miRNA by chondrocytes, Liang et al. introduced a chondrocyte-affinity peptide on the surface of exosomes and loaded the purified exosomes with miRNA-140 via electroporation [62]. Increased delivery efficacy to chondrocytes *in vitro* was revealed, which effectively attenuated the degeneration induced by inflammation. During an *in vivo* study, chondrocyte-targeted exosomes were injected into joint spaces and achieved a longer retention and deeper diffusion of miR-140 in a mouse model compared to non-targeted exosome vesicles [62]. Briefly, cell-specific exosomes enable themselves to be taken up by targeting cells more efficiently, which minimises cargo wastage during the transport process.

#### 2.2.4 Delivering exosomes through bioactive materials

Although the benefits of exosomes in cartilage regeneration are well established, the rapid diffusion and degradation of exosomes after injection into the cartilage defect can weaken the regenerative ability, placing restrictions on their extensive application [63]. Delivering exosomes through bioactive materials—which can not only retain the exosomes to achieve long-term release but also repair the defect using their bioactive components—is considered an available solution to this problem (Table 1). Injectable hydrogels made from different bioactive materials are some of the most attractive scaffolds enabling the long-term release of exosomes (Fig. 2). Acknowledging the importance of the sustained release of exosomes during *in vivo* application, Hu et al. developed a gelatin methacrylate/nanoclay hydrogel (Gel-nano) with outstanding biocompatibility, and mechanical and injectable properties for loading human umbilical cord MSC-Exos (hUC-MSCs-Exos) [63]. Exosomes could escape from this Gel-nano in a sustained manner while preserving a spherical microvesicle structure, indicating that the Gel-nano was a suitable vehicle for exosomes. In another study, miR-23a-3p was found to be the primary chondrogenic content in hUC-MSCs-Exo, and a Gel-nano with miR-23a-3p–abundant exosomes could facilitate cartilage restoration by activating the PTEN/AKT signalling pathway [63]. To adapt to the long healing process of cartilage, Liu et al. prepared a photo-induced imine crosslinking hydrogel glue as the container of exosomes to retain them at cartilage defect sites and exert their regenerative effect more durably. This novel hydrogel was available to preserve exosomes at >90% in 1 month, positively regulate both chondrocytes and BMSCs *in vitro* through exosome retention, and enhance cartilage formation in a rabbit model [64]. Recently, Zhang et al. also prepared an injectable adhesive hydrogel of alginate–dopamine, chondroitin sulphate and regenerated silk fibroin as a promising vehicle for MSC-Exos to achieve sustained release [32]. In an *in vitro* study using BMSCs, an adhesive hydrogel with encapsulated exosomes effectively recruited BMSCs, supported BMSC proliferation and guided them toward chondrocytes. Furthermore, the exosomes released from the novel adhesive hydrogel could promote the migration of endogenous BMSCs into the defect, which may be attributed to chemokine signalling pathways. Magnetic resonance imaging and histological analysis also indicated that the surface of regenerative cartilage was smoother and more similar to the native one with additional exosomes [32]. To prolong the effect of circRNA-enriched SMSC-Exos mentioned above, the researchers developed an injectable thermosensitive hydrogel using a poly(D,l-lactide)-b-poly(ethylene glycol)-b-poly(D,l-lactide) triblock copolymer as a vector. The novel hydrogel exhibited good performance in slowly releasing exosomes, effectively delivering circRNA3503-enriched exosomes to the chondrocytes and consequently protecting OA from progression *in vivo* [55].

In addition to hydrogels, decellularized cartilage matrix scaffolds also hold promise to be containers of exosomes. For instance, a recent study that implanted an acellular cartilage ECM (ACECM) scaffold into a cartilage defect found it could better retain the injected human Wharton’s jelly–derived MSC-derived exosomes (hWJMSC-Exos), aiming to promote the repair of rabbit osteochondral defect through the synergistic effect of decellularized scaffolds and exosomes [33]. The results showed that the cartilage defect managed by combining the ACECM scaffold and hWJMSC-Exo injection regenerated in a manner more complete and similar to the native cartilage compared to an exosome injection or scaffold alone. The applied hWJMSC-Exos were able to promote endogenous MSC proliferation and chondrogenic differentiation and to inhibit inflammation.

The biomimetic ACECM scaffold provides a suitable environment for endogenous MSC attachment, proliferation and chondrogenesis, improving cartilage regeneration with hWJMSC-Exos synergistically [33]. In addition to joint injection, MSC-Exos have been directly integrated with decellularized cartilage ECM and gelatin methacrylate (GelMA) to fabricate 3-dimensional scaffolds by desktop-stereolithography technology [45]. In a rabbit model with surgical osteochondral defects, incorporating exosomes into an ECM/GelMA scaffold significantly promoted cartilage regeneration owing to the effective restoration of cartilage mitochondrial dysfunction. Nevertheless, with regard to subchondral bone regeneration, additional exosomes in the ECM/GelMA scaffold were unable to improve the neo-bone volume or trabecular integrity [45].

Exosomes have also been injected with hyaluronic acid (HA), which can reduce inflammation and relieve joint pain, expecting to improve the microenvironment to restore cartilage defects. For instance, Wong et al. combined HA with MSC-Exos to repair osteochondral defects in rabbits. After 12 weeks, the defects treated by HA and MSC-Exos were restored by hyaline cartilage with superior mechanical properties, while those treated by HA alone showed deteriorated cartilage formation [65]. A recent study employing a porcine model showed that osteochondral defects treated by intra-articular injections of MSC-Exos and HA simultaneously attained improved morphological, histological and biomechanical outcomes compared to HA-treated lesions [66]. These results provide a powerful scientific basis for clinical translation of co-administration of exosomes and injectable bioactive materials for cartilage repair in OA.

Collectively, biomimetic materials are outstanding holders to preserve and protect exosomes at the cartilage defect, enabling exosomes to display long-term regenerative efficacy. The combination of exosomes and bioactive materials can efficiently regenerate cartilage defects in OA by recruiting endogenous chondrocytes, promoting their proliferation by exosomes and providing a favourable microenvironment through bioactive materials. Moreover, exosomes also hold promise to attract endogenous MSCs and guide them toward a chondrogenic lineage, effectively renewing aged chondrocytes with a degenerative status.

## 3 Exosome-based strategies for IVDD

### 3.1 Native exosome application in IVDD

#### 3.1.1 Promotion of nucleus pulposus cell proliferation, migration, differentiation and anti-senescence

**Fig. 3:**
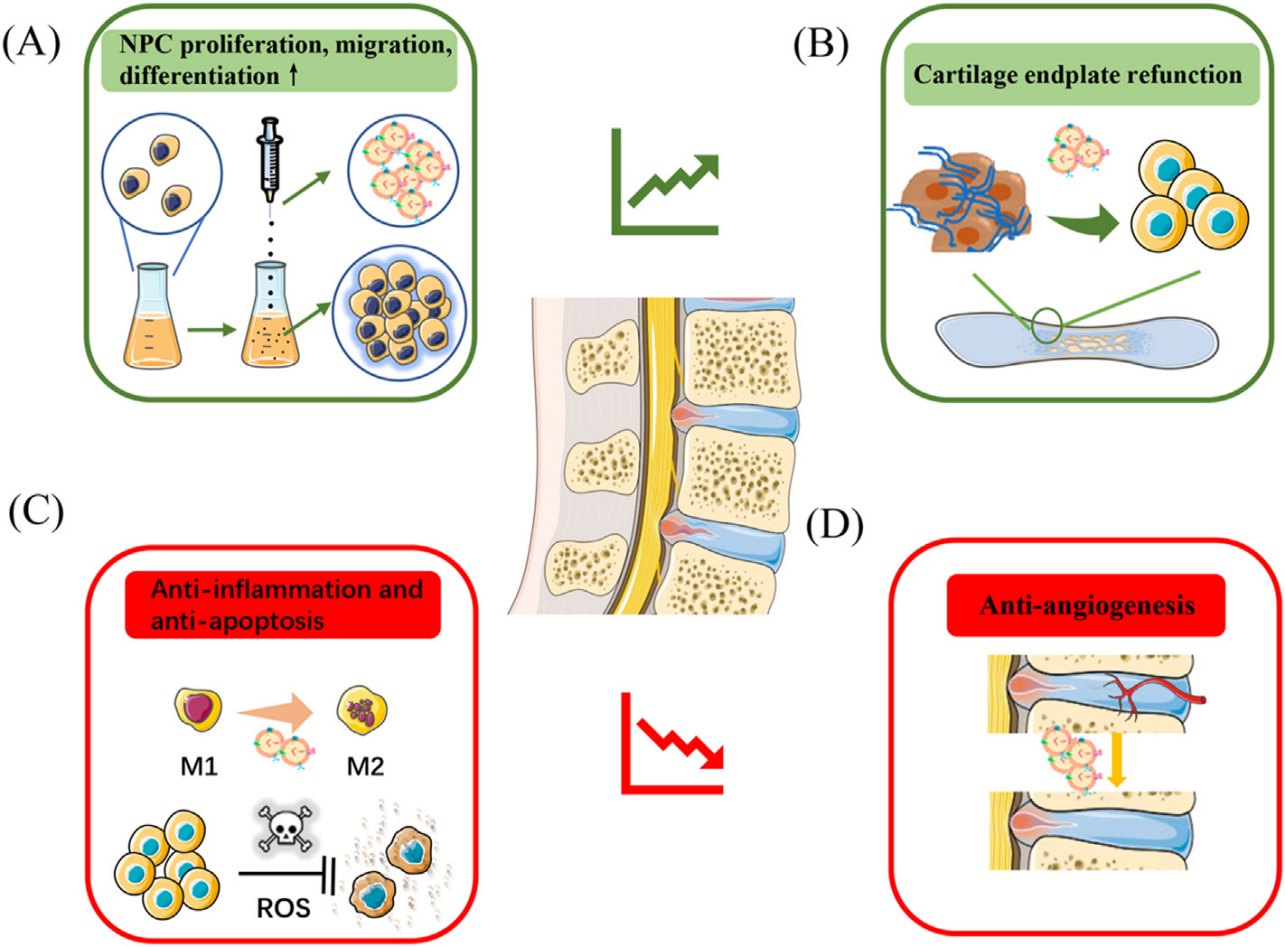
Illustration of the functions that native exosomes exert in intervertebral disc degeneration: (A) Promotion of nucleus pulposus cells proliferation, migration and differentiation. (B) Cartilage endplate refunction. (C) Anti-angiogenesis. (D) Anti-inflammation and anti-apoptosis.

A gradual reduction in functional nucleus pulposus cells (NPCs) is considered to be the main pathological change in IVDD, resulting in an imbalance between ECM synthesis and degradation [67]. One of the promising options to combat this is a cell-free therapy strategy to recruit stem cells to the injured site and induce them towards NPCs (Fig. 3A). For instance, Lu et al. reported that NPC-secreted exosomes could effectively improve the migration of MSCs in Transwell assays as well as induce MSC differentiation to a nucleus pulposus-like phenotype as evidence of higher NPC phenotypic gene expression [68]. MSCs-Exos also hold promise to reactivate degenerative NPCs for healthier ECM production, providing an alternative to overcome the challenge of reduced functional NPCs in IVDD [68]. Recently, Guo et al. revealed that urine stem cell-derived exosomes (USC-Exos) enabled the attenuation of IVDD by exosomal MATN3. Exosomal MATN3 could effectively promote NPC proliferation and exert anti-senescence effects, improving IVDD in mice as evidence of better disc height preservation as measured by computed tomography and lower degenerative scores as assessed by magnetic resonance imaging [69]. Additionally, USC-Exos possessed the strength of a wide range of sources, are convenient to obtain, have relatively safe and non-invasive characteristics and do not have ethics issues, making them promising potential candidates for exosome extraction [69]. It has also been reported that exosomes derived from cartilage endplate stem cells (CESC-Exos) can induce stem cells originating from the cartilage endplate to migrate into the intervertebral discs and differentiate to NPCs through hypoxia-inducible factor 1α/Wnt pathway activation, helping young cells to overcome aging-related degeneration and achieving tissue repair [70]. To clarify the functional components mediating the therapeutic effect of native exosomes in IVDD and their relevant molecular pathways, a summary is provided in Table 1.

#### 3.1.2 Refunction of cartilage endplate

As the degeneration of the cartilage endplate has been proved to exacerbate IVDD by disturbing nutrient transportation, priority should be given to restoring supplementation from the cartilage endplate to NPCs [71]. One of the available strategies to achieve this is supplying CESC-Exos to degenerative NPCs, which was performed by Luo et al. in IVDD rat models (Fig. 3B). Their results showed CESC-Exos could attenuate IVDD by apoptosis inhibition due to activation of the PI3K/AKT/autophagy pathway [72]. Another therapeutic strategy is to inhibit the degeneration of endplate chondrocytes, as Xie et al. indicated that MSC-Exos could attenuate the apoptosis and calcification of endplate chondrocytes in a tert-butyl hydroperoxide–induced oxidative stress model [73]. In their *in vivo* experiment, not only NPCs but also endplate chondrocytes were better preserved after the administration of MSC-Exos. The exosomal miR-31-5p targeting apoptosis-related ATF6 is regarded as the underlying mechanism for the favourable effect of MSC-Exos in reversing cartilage endplate degeneration [73].

#### 3.1.3 Anti-inflammation, anti-pyroptosis and anti-apoptosis

Similar to OA, inflammation and oxidative stress induced by accumulated advanced glycation end-products with aging are partly responsible for IVDD progression [74,75]. The release of inflammatory cytokines could mediate apoptotic and pyroptotic cell death and ECM destruction, resulting in biomechanical and structural deterioration [76,77]. Thus, an anti-inflammatory capacity would be of vital importance in treating IVDD (Fig. 3C). It has been reported that MSC-Exos play an anti-oxidant and anti-inflammatory role in preventing the progression of IVDD, which was explained by NLRP3 inflammasome inactivation and damaged mitochondria restoration [78]. The NLRP3 pathway could also be suppressed by exosomal miR-410, down-regulating the pyroptosis of NPCs in IVDD [79]. More importantly, Zhu et al. suggested that MSC-Exos pre-treated with TNF-α could better inhibit the apoptosis of NPCs under inflammatory microcircumstances compared to those that remained untreated [80]. Moreover, a recent study revealed that human umbilical cord MSC exosomes could target METTL14, which stabilised NLRP3 messenger RNA in an IGFBP2-dependent manner through exosomal miR-26a-5p, protecting NPCs from pyroptosis caused by inflammation [81]. MSC-Exos have also been demonstrated to protect NPCs from apoptosis via miR-21 and miR-142-3p within exosomes, achievements which were attributed to PTEN/PI3K/Akt pathway silencing and MAPK signalling pathway suppression, respectively [82,83]. Moreover, excessive endoplasmic reticulum stress in IVDD has also been verified as a crucial mediator of NPC apoptosis [84]. It was proved that MSC-Exo therapy could also massively reduce endoplasmic reticulum stress, thus inhibiting the apoptosis of NPCs [84]. In addition to MSC-Exos, USC-Exos can also alleviate endoplasmic reticulum stress on pressure-induced NPC apoptosis while holding the advantages of non-invasion and high convenience compared to MSC-Exos, opening the possibility of the clinical application of exosomes in IVDD [85].

#### 3.1.4 Anti-angiogenesis

In normal intervertebral disc, blood vessels are limited in the outer surface of the annulus fibrosus, while ingrowth of blood vessels was observed in the context of IVDD, disrupting the maintenance of normal disc homeostasis and function [86]. Because angiogenesis is the pathological signature of IVDD, an anti-angiogenesis capacity is also indispensable to slow or halt the degeneration of nucleus pulposus (Fig. 3D). Recently, Sun et al. revealed that notochordal cell–derived exosomes (NC-Exos) were able to prevent angiogenesis by delivering miR-140-5p to endothelial cells and consequently regulating the Wnt/β-catenin pathway [87]. This mechanism was also consistent with the clinical evidence that IVDD patients with worsened angiogenesis were prone to showing lower exosomal miR-140-5p expression in the nucleus pulposus [87]. Noticeably, a 0.5-MPa compressive load could improve the anti-angiogenesis ability of NC-Exos through simulating mechanical stresses in the normal environment of NC survival and function. Overall, NC-Exos were able to inhibit angiogenesis in vascular epithelial cells to alleviate IVDD, providing a novel target for IVDD therapy in the future.

### 3.2 Modified exosome–based strategies for IVDD

Currently, the use of modified exosome–based therapy in IVDD is relatively rare. Exosomes are most commonly used to deliver exogenous miRNA to attenuate the progression of IVDD. A reduction in the amount of NPCs is considered to be responsible for the initiation of IVDD [88,89]. Although MSCs have recently been identified in NP tissue, they seldom differentiate into NPCs under normal circumstances [90], indicating that exosomes with endogenous miRNA might be insufficient to guide stem cells towards NPCs. To promote chondrogenic differentiation of NP-MSCs, NP-MSC–derived exosomes with exogenous miR-15a, which are known to regulate cell proliferation and apoptosis, were developed to repair chondral defects [91]. The results indicated that exosomes with abundant miR-15a successfully guided NP-MSCs towards a chondrogenic lineage via interacting with matrix metalloproteinase 13 [91]. MiR-21, the microRNA down-regulated in apoptotic NPCs, was also delivered by purified MSC-Exo to alleviate IVDD degeneration. The results indicated that intradiscal injection of MSC-Exo with abundant exogenous miR-21 significantly inhibited the apoptosis of NPCs and attenuated IVDD progression [82].

Given the rapid clearance and disruption of exosomes in IVDD therapy, it is necessary to find a vehicle that enables long-term exosome release *in vivo*. Decellularized scaffolds are currently regarded as one of the most suitable bioactive materials to load with exosomes to achieve sustained release because of their appropriate degradation, allowing exosomes to remain in the disc for a long time [92,93]. Moreover, decellularized scaffolds could also exert a synergistic effect with exosomes to provide a favourable microenvironment for NPCs [94]. Recently, Xing et al. developed a thermosensitive acellular ECM hydrogel to transport MSC-Exos to degenerated vertebral discs, providing an environment for the growth of NPCs [92] (Table 2). This novel hydrogel could also fill the defects resulting from ECM catabolism through *in situ* gelation. The designed acellular hydrogel with exosomes effectively protected NPCs from pyroptosis *in vitro* and delayed IVDD in a mouse model, which was attributed to the co-operation of acellular scaffolds and exosomes [92].

**Table 2:** Summary of bioactive materials utilized to deliver exosomes in osteoarthritis and intervertebral disc degeneration.

Bioactive materials	Exosomes derived	Usage method	Application	In vitro	In vivo	Rf
Gel-nano hydrogel	hUC-MSCs	injection	OA	BMSCs/chondrocytes	rat	[63]
HA-NB hydrogel	hiPSC-MSCs	injection	OA	BMSCs/chondrocytes	rabbit	[64]
AD-CS-RSF hydrogel	BMSCs	injection	OA	BMSCs	rat	[32]
Triblock copolymer gel	SMSCs	injection	OA	chondrocytes	rat	[55]
aECM hydrogel	ADSC	injection	IVDD	NPCs	rat	[92]
ACECM scaffold	hWJMSCs	implantation	OA	BMSCs/chondrocytes	rabbit	[33]
ECM/GelMA scaffold	BMSCs	implantation	OA	chondrocytes	rabbit	[45]
HA	hESCs-MSCs	injection	OA	/	rabbit/pig	[65,66]

Gel-nano, Gelma/nanoclay; hUC-MSCs, human umbilical cord mesenchymal stem cells; OA, osteoarthritis; HA-NB, o-nitrobenzyl alcohol moieties modified hyaluronic acids, hiPSC-MSCs, human induced pluripotent stem cells-derived MSCs; AD-CS-RSF, alginate-dopamine, chondroitin sulfate, and regenerated silk fibroin; SMSCs, synovium mesenchymal stem cells; ADSC, adipose-derived mesenchymal stem cell; IVDD, intervertebral disc degeneration; NPCs, nucleus pulposus cells; aECM, acellular extracellular matrix; ACECM, acellular cartilage extracellular matrix; hWJMSCs, human Wharton’s jelly-derived MSCs; GelMA, gelatin methacrylate; HA, hyaluronic acid; hESCs-MSCs, human embryonic stem cell-derived MSCs.

## 4 Conclusion and perspective

Exosome therapy has progressed significantly in recent years and is widely accepted as one of the most potent strategies for treating degenerative diseases in their early stage. Previously, exosomes were directly injected at the degenerative site in their native status after being extracted from various stem cells, such as MSCs and chondrocytes. Nevertheless, the injection of native exosomes showed several disadvantages, including rapid diffusion, disruption and fluctuating functional contents, discouraging their application in some complex healing processes. In order to better match the requirements of the regenerative process, various modified exosome–based strategies have been developed. Given the fluctuating and insufficient functional contents of native exosomes, several exogenous small molecules, including miRNAs, circRNAs and proteins, were integrated into exosomes to further improve the anti-senility capacity in degenerative orthopaedic diseases. With regard to the rapid clearance and disruption of exosomes, researchers have developed bioactive scaffolds to deliver exosomes, which could not only release exosomes in a sustained fashion but also repair the degenerative tissue synergistically. Furthermore, exosomes with specific sequences targeting certain cells were also designed to achieve high transport efficacy and long-term maintenance in the degenerative tissue.

Aging is inevitable, and senility is often accompanied by degenerative disease, badly affecting life quality. Currently, no approved therapeutic agents are available to reverse or counteract the progression of degenerative disease in orthopaedics. Conservative therapies, including self-management and education, physiotherapy and pain control, are the first-line treatment strategy for OA and IVDD [[95], [96], [97]]. When a disease reaches its end-stage, surgical procedures become the only practical option [95,98]. One of the most significant barriers to the development of therapeutic strategies to combat orthopaedic degenerative disease is an unclear and complex pathogenesis. However, recent studies indicate that the circulating extracellular vesicles from young serum could rejuvenate aged cell bioenergetics to promote brain and skeletal muscle regeneration in aging animals, opening the possibility of treating degenerative diseases using novel exosome-based therapies [99,100]. In addition to its extraordinary therapeutic effect, exosome-based therapy also shows the advantages of a high feasibility of clinical translation; indeed, the first-in-human application of MSC-derived EVs was recently reported in Australia [101]. Considering the non-invasive properties of injection, as well as the biocompatibility of exosomes, injectable hydrogels with loaded exosomes or exosomes with small molecular drugs hold promise to manage OA and IVDD prior to patients reaching the advanced stage of disease in the future.

Exosome-based therapy is currently a hot research topic promising tissue restoration in the context of degenerative conditions. Exosomes enable tissue regeneration not only in degenerative diseases of orthopaedics but also retinal and even nervous system degeneration [102,103]. However, there are still many challenges that should be addressed before wide clinical application of exosomes. One of the most concerning obstacles is that the efficiency of exosome preparation is limited by the production capacity of the current cell-expansion regime. The improvement of current protocols is urgently required to enable cost-effective, industrial-scale production of exosomes [104]. Other resources besides stem cells to acquire exosomes in bulk and cost-effectively could also be helpful in the clinical translation of exosome-based therapy. For instance, bovine milk might serve as a scalable source to allow safe and cost-effective production of exosomes in bulk [105]. Another challenge in the clinical translation of exosomes is the concern of safety. Although the efficacy of exosomes for degenerative diseases such as OA and IVDD has been well investigated, studies focusing on the safety of exosomes are relative scarce. Moreover, the efficacy and safety of exosome-based strategies should also be confirmed in a larger animal model possessing a microenvironment resembling that of humans. Additionally, a considerable number of studies found that the tissue repaired by exosomes is still far less than the native tissue, indicating that the combination of exosomes and other therapeutic strategies should be attempted to restore degenerated tissue more comprehensively. Other limitations of exosomes, including their rapid diffusion and degradation, low purity and weak targeting, also hinder their clinical application, although these limitations have already been partially overcome by modified exosome–based strategies. Collectively, exosome-based therapy shows a huge potential to reverse degenerative orthopaedic disease in the future, but there is still a long way to go for its clinical translation.

## Declaration of competing interest

The authors have no conflicts of interest to disclose in relation to this article.
